# Construct validity of measures of care home resident quality of life: cross-sectional analysis using data from a pilot minimum data set in England

**DOI:** 10.1186/s12955-025-02356-0

**Published:** 2025-04-05

**Authors:** Stephen Allan, Stacey Rand, Ann-Marie Towers, Kaat De Corte, Freya Tracey, Elizabeth Crellin, Therese Lloyd, Rachael E. Carroll, Sinead Palmer, Lucy Webster, Adam Gordon, Nick Smith, Gizdem Akdur, Anne Killett, Karen Spilsbury, Claire Goodman

**Affiliations:** 1 Personal Social Services Research Unit (PSSRU), University of Kent, Kent, UK; 2 Health and Social Care Workforce Research Unit, King’s College London, London, UK; 3 The Health Foundation, London, UK; 4 School of Medicine, University of Nottingham, Nottingham, UK; 5 Centre for Health Services Studies (CHSS), University of Kent, Kent, UK; 6 Queen Mary University, London, UK; 7 Centre for Research for Public Health and Community Care, University of Hertfordshire, Hatfield, AL10 9AB UK; 8 School of Health Sciences, University of East Anglia, Norwich, NR4 7TJ UK; 9 School of Healthcare, Faculty of Medicine and Health, University of Leeds, Leeds, UK

**Keywords:** Quality of life, Measurement, Care homes, Older adults, England, Validity.

## Abstract

**Background:**

To maintain good standards of care, evaluations of policy interventions or potential improvements to care are required. A number of quality of life (QoL) measures could be used but there is little evidence for England as to which measures would be appropriate. Using data from a pilot Minimum Data Set (MDS) for care home residents from the Developing resources And minimum dataset for Care Homes’ Adoption (DACHA) study, we assessed the discriminant construct validity of QoL measures, using hypothesis testing to assess the factors associated with QoL.

**Methods:**

Care home records for 679 residents aged over 65 from 34 care homes were available that had been linked to health records and care home provider data. In addition to data on demographics, level of needs and impairment, proxy report measures of social care-, capability- and health-related QoL of participants were completed (ASCOT-Proxy-Resident, ICECAP-O, EQ-5D-5L Proxy 2). Discriminant construct validity was assessed through testing hypotheses developed from previous research and QoL measure constructs. Multilevel regression models were analysed to understand how QoL was influenced by personal characteristics (e.g. sex, levels of functional and cognitive ability), care home level factors (type of home, level of quality) and resident use of health services (potentially avoidable emergency hospital admissions). Multiple imputation was used to address missing data.

**Results:**

All three QoL measures had acceptable construct validity and captured different aspects of QoL, indicated by different factors explaining variation in each measure. All three measures were negatively associated with levels of cognitive impairment, whilst ICECAP-O and EQ-5D-5L Proxy 2 were negatively associated with low levels of functional ability. ASCOT-Proxy-Resident was positively associated with aspects of quality and care effectiveness at both resident- and care home-level.

**Conclusion:**

The study found acceptable construct validity for ASCOT-Proxy-Resident, ICECAP-O and EQ-5D-5L Proxy 2 in care homes, with findings suggesting the three are complementary measures based on different constructs. The study has also provided evidence to support the inclusion of these QoL measures in any future MDS.

**Supplementary Information:**

The online version contains supplementary material available at 10.1186/s12955-025-02356-0.

## Background

Although global policy is progressively shifting towards provision of care in the community, many people still live in care homes. In England, around 315,000 people aged 65 and over live in care homes [1]. Limited budgets means that decisions need to be made, not only on where care is received, but also on how to maintain standards of care for people living in care homes, ensuring good quality of life (QoL) outcomes. Evaluations of policy or potential improvement in care are required, including economic evaluations.

An important outcome measure for care home residents is their QoL, which can be appropriately assessed using several measures. A systematic review of QoL measures found 14 instruments had been evaluated psychometrically with a care home population [2]. In terms of economic evaluation, it is ideal if these measures can be converted, along with knowledge of life expectancy, into quality-adjusted life years (QALYs), or an equivalent [3]. The most widely-used measure of this kind is the EuroQol five-dimension questionnaire (EQ-5D), a measure of health-related quality of life [4]. However, given the aim of care is to support residents’ QoL, beyond health, it is important to also consider other measures of broader QoL for use in care home economic evaluations [5]. These include the Adult Social Care Outcomes Toolkit (ASCOT) and the ICEpop CAPability measure for older people (ICECAP-O). ASCOT is a suite of social care-related QoL measures and is suitable for use in care homes [6–8]). ICECAP-O is a measure of QoL from a capability wellbeing perspective, assessing whether a person can do the things that are important to them in life [9–10]. ASCOT, which has been developed to be responsive to changes in social care delivery, and ICECAP-O, which looks at broader aspects of life in general for older people, may be preferable to EQ-5D for evaluations of care home interventions, as they could have greater sensitivity to QoL changes attributable to social care [11–12]. Ultimately, the three measures described above already have research evidence as to their responsiveness in detecting clinically important intervention changes [2] and the value of using one QoL measure over another may depend on the focus of evaluation, e.g. health, social care, or life in general [13–15]. However, these studies, with a few exceptions [8, 16], have tended to focus mainly on data collection from older people living at home, accessing home care or community-based social care services.

ASCOT-Proxy-Resident, the proxy report version of ASCOT used in this study, has been found to have good structural validity and evidence of consistency with ASCOT-SCT4, the version of ASCOT upon which it is based [17]. Further, care home resident QoL in England, when measured using ASCOT, is positively associated with resident cognitive functioning, care home quality ratings and weakly with resident functional ability [7, 16, 18]. The latter finding was expected given the instrument measures social care-related QoL, and, as such, a person’s (in)ability to achieve activities of daily living (ADLs) should be accounted for by the level of care they receive. ICECAP-O has good construct validity and responsiveness for older people in general [3]. Convergent and discriminant validity of the measure has been established for nursing home residents, internationally, with both functional and cognitive ability associated with the measure [19–21]. However, it has only been used in one previous study including residents in English care homes [22], with limited evidence on its validity [23]. EQ-5D has good validity for use in care homes [2] and a recent study found adequate feasibility and validity for the proxy report version of EQ-5D used in this study, EQ-5D-5L Proxy 2, in Singaporean nursing homes [24]. In England, another proxy version, EQ-5D-5L Proxy 1, which asks the proxy respondent to rate a person’s health, rather than asking the proxy to rate how they think the person would rate their own health (as for EQ-5D-5L Proxy 2), has been found to have good validity [25]. The same study found it was also responsive to changes in functional and cognitive impairment when used in care homes by staff. Internationally, measures of EQ-5D have associated QoL with functional ability [19, 21], but not cognitive impairment [26–27]. These latter findings indicate the potential difficulty of using a measure of health-related QoL in a care home setting.

For care homes, there is emerging evidence from a parallel study of the feasibility and construct validity of the three QoL measures used in this study, ASCOT-Proxy-Resident, ICECAP-O and EQ-5D-5L Proxy 2 [28]. The latter was assessed using hypothesis testing of correlations between the measures, i.e. convergent construct validity. However, more evidence is required as to the validity of using these three QoL measures in care homes in England, and, importantly, in understanding what factors, such as needs, demographic characteristics and health care utilisation, influence care home resident QoL. As outlined above, there are may be important differences in what causes variation in QoL depending on the instrument used to assess QoL. For older people, there is evidence on comparative differences between QoL instruments [2, 11, 13, 29], but evidence for care home residents in England is limited.

Studies have also not assessed the influence of health care utilisation on care home residents’ QoL in England. Care home residents’ QoL is likely to be affected by their health conditions [18, 20, 30–31] and health care use, including hospital admissions deemed preventable [32–33]. Avoidable hospital admissions are a major area of concern for policymakers, and particularly so for care home residents, with 185,000 emergency admissions each year and up to 40% of these potentially avoidable [34]. They are also of interest to care home residents [35]. International studies have associated hospitalisation to both unmet need [36–37] and the quality of care received [38–39].

Given all of the above, the aim of this study was to assess the construct validity of three QoL instruments (ASCOT-Proxy-Resident, ICECAP-O and EQ-5D-5L Proxy 2) for use by staff report in care homes using discriminant analysis and, in so doing, increase understanding of the factors associated with care home resident QoL. Discriminant construct validity was assessed by hypothesis testing utilising multi-level model regression analysis, using data from a pilot minimum data set (MDS) developed as part of the Developing resources And minimum data set for Care Homes’ Adoption (DACHA) study [40–44]. The pilot MDS linked care home digital care records (DCRs) with both health care records and other relevant administrative data [41]. The MDS data included personal (e.g. sex, level of functional need, QoL and cognitive impairment) and care home (e.g. type and quality rating) characteristics. The data also included measures of health care utilisation, such as potentially avoidable emergency hospital admission. Therefore, along with providing further evidence of the validity of these three QoL measures in care homes, the study also adds to the current evidence base for England of the factors related to resident QoL outcomes.

## Methods

This study is reported using the Strengthening Reporting of Observational Studies in Epidemiology (STROBE) cross-sectional reporting guidelines [45].

### Study design and participants

From three areas of England, the study recruited 996 residents from 45 care homes (residential and nursing homes) that used digital care planning software from two providers. All permanent residents were eligible to be included in the study with the exception of those that were at the end-of-life, as judged by care home staff. Study design and participant recruitment are reported in detail elsewhere [41, 44].

The Pilot MDS (wave 1) contained data for 727 residents from 38 care homes [41]. Forty-eight of these residents (6.6%) were excluded from analysis because either their record did not include a valid Care Quality Commission (CQC) care home ID to indicate where they lived, which was required for matching to care home level data (*n* = 31) or they were under the age of 65 (*n* = 17). Analysis proceeded using data for 679 residents from 34 care homes, referred to herein as ‘the study sample’. The study sample was intended to be representative of the areas that participating care homes were located [44], but we could not confirm this with available data. Instead, we assessed the national representativeness of the residents in the study sample to the 2021 care home resident population aged over 65 [1].

### Dependent variables

In addition to existing digital care records on personal demographics and needs, several QoL measures were selected for inclusion in the pilot MDS [40]. In particular, data were collected through staff proxy response for ASCOT-Proxy-Resident and ASCOT-Proxy-Proxy (social care-related QoL), ICECAP-O (capability wellbeing), QUALIDEM (dementia-specific QoL), and EQ-5D-5L Proxy 2 (health-related QoL). For this analysis, three QoL measures, ASCOT Proxy-Resident, ICECAP-O and EQ-5D-5L Proxy 2, were considered because there is evidence supporting their construct and structural validity, internal consistency and feasibility in older adult care home data collections, both in England and internationally [17, 19, 24, 28].

#### Ascot-Proxy-Resident

ASCOT-Proxy is a questionnaire collecting data for two separate measures of social care-related QoL (SCRQoL) [46]. It covers eight social care domains: personal comfort and cleanliness, personal safety, food and drink, activities/occupation, control over daily life, social participation, home cleanliness and comfort, and dignity, with four levels (ideal state, no needs, some needs, and high needs). Proxy respondents (in this study, care home staff) are asked to rate ASCOT-Proxy items from both the proxy-resident (i.e. what the proxy thinks the resident thinks) and proxy-proxy (i.e. what the proxy thinks about the resident’s QoL) perspective. Two measures of proxy-report social care-related QoL are generated: ASCOT-Proxy-Resident and ASCOT-Proxy-Proxy. ASCOT-Proxy-Resident was included in this analysis because it has been found to be a valid QoL instrument, with the same structure as the original ASCOT-SCT4 measure from which it was adapted, for data collected with proxy respondents for care home residents [17]. An index score (-0.171 to 1) was generated using preference weights for ASCOT-SCT4, with 0 being equivalent to ‘being dead’ and 1 representing the ideal social care-related QoL state [6]​​.

#### ICECAP-O

ICECAP-O is a measure of capability wellbeing [10, 47]. ICECAP-O has five items: attachment, security, role, enjoyment, and control, with four levels of response that represent capability (none, a little, a lot, and all). There is no formally adapted proxy report version of the ICECAP-O, although ICECAP-O has been informally adapted for proxy report by staff in care homes [19]. In this study, ICECAP-O was collected by proxy report using the standard ICECAP-O questionnaire, without adaptation. The score (0 to 1) was calculated using UK index values [10], ranging from 0 (no capability) to 1 (full capability). International research has recommended the use of the ICECAP-O in care homes via staff proxy report [19] and initial evidence for England supports this [28].

#### EQ-5D-5L proxy 2

The EQ-5D-5L measures individuals’ level of functioning in five domains: pain, mobility, usual activities, anxiety/depression, and self-care, with five levels (no problems, slight problems, moderate problems, severe problems, and extreme problems) [48–49]. In this study, we used the EQ-5D-5L Proxy 2 version, which asks proxy respondents (care staff) to rate QoL from the proxy-resident perspective. The EQ-5D-5L score (-0.594 to 1) was calculated using the mapping function to convert to EQ-5D-3L and applying its UK index values since the UK value set for EQ-5D-5L is still being developed [50–51]. A score of 1 represents full health and 0 is an equivalent state to death.

### Independent variables

The pilot MDS contained data on a number of factors likely to be associated with QoL which were included in the analysis. The following measures of personal function were included: functional independence (Barthel Index), cognitive impairment (MDS Cognitive Performance Scale, MDSCPS) and delirium (Informant Assessment of Geriatric Delirium Scale, IAGeD) [52–54]. Length of stay was calculated using date of admission from care home records. Sex, age, and ethnicity were available from health records. Given the very small number of residents that were not in the White high-level ethnic grouping (*n* = 8), we excluded ethnicity from the regression analysis. Health care records also included information on admissions and medical conditions. Comorbidities was measured if a resident had two or more Elixhauser comorbidities during hospital admissions in the previous three years [55–56], and potentially avoidable emergency hospital admissions in the previous 12 months was included in the study as an indicator of unmet needs. A potentially avoidable admission was where the primary diagnosis was a condition often considered manageable, treatable or preventable in community settings, or that may be caused by poor care or neglect [57]. Finally, we included the following factors at care home-level: type of home (residential or nursing), number of beds, occupancy rate, self-funding rate (i.e. percentage of residents funding their own stays) and most recent CQC quality rating (‘Inadequate’, ‘Requires improvement’, ‘Good’ or ‘Outstanding’).

### Hypotheses

To establish discriminant validity, we used hypothesis testing by utilising multi-level model regressions, controlling for personal and care home characteristics described above. We tested for differences in QoL score between residents dependent on characteristics. Resident-level independent variables were recoded into categories based on previous research and measure constructs [16, 20, 54].

Table 1 presents the hypotheses, which were based on previous research or a priori informed by the measurement constructs. Sufficient evidence of construct validity was considered using a criterion of > = 75% of hypotheses accepted for each QoL instrument [58].

**Table 1:** Discriminant construct validity hypotheses, by QoL measure

QoL instrument	Variable	Expected association	Hypothesis accepted
ASCOT-Proxy-Resident	Functional dependence	No significant (or significant low negative) association based on previous research with care home residents in England [8, 16, 18, 28].	Y
	Cognitive impairment	Significant negative association based on previous research with care home residents in England [8, 16, 18, 28].	Y
	Potentially avoidable emergency hospital admission	Significant negative association based on measurement construct, which is designed to capture quality and effectiveness of care [6, 46].	Y
	Care home quality	Significant positive association based on previous research with care home residents in England [14–15] and measurement construct (i.e. SCRQoL), which is designed to capture the quality and effectiveness of care [6, 46].	Y
ICECAP-O	Functional dependence	Significant negative association based on previous research with care home residents internationally [19–21].	Y
	Cognitive impairment	Significant negative association based on previous research with care home residents internationally [19–21].	Y
	Potentially avoidable emergency hospital admission	No significant association based on measurement construct, which is designed to capture capability wellbeing rather than quality and effectiveness of care [9–10, 47].	Y
	Care home quality	No significant association based on measurement construct, which is designed to capture capability wellbeing rather than quality and effectiveness of care [9–10, 47].	Y
EQ-5D-5L Proxy 2	Functional dependence	Significant negative association based on previous research with care home residents internationally [16, 18] and measure construct, which includes a rating of ‘usual activities’. This overlaps with the construct of functional independence, as measured by the Barthel Index [48–49, 52].	Y
	Cognitive impairment	No significant association based on previous research with care home residents internationally [26–27].	N
	Potentially avoidable emergency hospital admission	No significant association based on instrument construct, which is not designed to capture care quality and effectiveness [48–49].	Y
	Care home quality	No significant association based on instrument construct, which is not designed to capture care quality and effectiveness [48–49].	Y
All QoL measures	Functional dependence	EQ-5D-5L Proxy 2 will have a stronger sized negative association than ICECAP-O, and ICECAP-O a stronger sized negative association than ASCOT-Proxy-Resident, based on measure constructs [6, 9–10, 46–49].	Y

### Statistical methods

#### Missing data strategy

There was missing data in the MDS. We confirmed that the data were not missing completely at random (MCAR) using logistic regression of binary missing data indicators for each QoL measure. Therefore, we assumed the data were missing at random (MAR) and used multiple imputation (MI) to address the missing data given a complete case analysis could provide inefficient and biased estimates [59]. The QoL models were multi-level (see below). As such, we imputed the data using the chained imputation method with predictive mean matching (QoL measures, age, length of stay, Barthel index, IAGeD, occupancy rate, self-funding rate, staff-resident ratio), Poisson (number of comorbidities) and ordered logistic (MDSCPS) models at two levels, care home and resident [60–61]. In the first imputation step, we imputed missing care home-level data (ten imputations), such as occupancy and self-funding rates, with the imputation process including mean data of known resident characteristics [60, 62]. We then included each care home-level imputation individually in the imputation of data at the resident-level (ten imputations). This generated a resident-level dataset with 100 imputations. We conservatively chose the number of imputations to provide adequate levels of reproducibility (i.e., the same results would be found if the multiple imputation was repeated). To confirm reproducibility of the findings, we assessed the random errors generated by the MI process in the estimations of QoL [63].

#### Regression model

To allow for factors that could influence the variation in QoL at resident- and care home-level, we estimated a ‘within-between’ multi-level model of each QoL measure, with care home residents clustered by care home [64] and categorical variables included in models using dummy codes [65]. The ‘within-between’ multi-level model separates out level 1 (i.e., resident) associations into within and between associations. Resident-level predictors are demeaned (care home means) to determine within-care home associations, i.e. differences in QoL between residents based on characteristics, and the care home means of resident-level predictors are separately included at level 2 (i.e. care home level) to capture between-care home associations, i.e. differences in QoL between care homes based on average resident characteristics. At the same time, this method allows for appropriate estimation of the association of other care home characteristics with QoL and controls for omitted variables at the care home-level (e.g. location).

Given MI, the appropriateness of the multi-level structure was assessed pragmatically with Likelihood-ratio tests of the null hypothesis of no variance between care homes for each imputation. Standard errors were clustered by care home.

We carried out the analysis using Stata 18 and set a statistical significance using two-sided tests of 0.05.

## Results

### Descriptive statistics

Representativeness of the study sample to the overall care home resident population of England is presented in Table 2. The study sample was similar to the resident population by sex and type of care home but overrepresented over 85s and the White high-level ethnic grouping.

**Table 2:** Comparison of study sample to English care home resident population

		Study sample	Care home resident population	Study sample v population^a^
Age 85 and over (%)		60.2	56.4	2.02*
Non-white (%)		1.2	2.5	-2.11*
Male (%)		28.9	30.3	-0.81
Nursing home (%)		51.8	49.4	1.27

Source: Care home resident population data from 2021 census data [1]

^a^Hypothesis test of equality of the study sample proportion for a characteristic to the care home resident population. *p <.05

Table 3 presents descriptive data of the residents in the study sample, including levels of missing data. The complete data showed that the average resident had an ASCOT-Proxy-Resident, ICECAP-O and EQ-5D-5L Proxy 2 score of 0.831, 0.738 and 0.342, respectively. There was a high level of dependence with activities of daily living amongst the residents, as 70% of residents did not have functional independence, whilst cognitive impairment as assessed by MDSCPS ranged from no impairment (19.0%) to severe or very severe impairment (25.4%). A number of residents (16.3%) had a potentially avoidable emergency hospital admission in the previous year. The residents lived in 34 care homes, 19 of which were nursing homes, with slightly more residents living in the latter (51.8%). Almost three quarters of residents lived in a care home rated as ‘Good’ (73.2%), with 16.3% and 10.5% living in homes rated as ‘Requires improvement’ and ‘Outstanding’, respectively. The average resident lived in a care home with 54 beds, an 88.0% occupancy rate and with 49.0% of residents self-funding, i.e. paying for their own care.

**Table 3:** Descriptive statistics of care home residents in study sample (*n* = 679)

		*n* (% missing)	Value
**Resident-level characteristics**			
ASCOT Proxy-Resident: mean (s.d.)		454 (33.1)	0.831 (0.188)
ICECAP-O: mean (s.d.)		527 (22.4)	0.738 (0.202)
EQ-5D-5L Proxy 2: mean (s.d)		584 (14.0)	0.342 (0.354)
*Age*		675 (0.6)	
65–79		135	20.0%
80–89		306	45.3%
90+		234	34.7%
*Sex*		679 (0.0)	
Female		483	71.1%
Male		196	28.9%
*Length of stay*		679 (0.0)	
Length of stay: < 3 years		497	73.2%
Length of stay: >=3 years		182	26.8%
*Functional independence (Barthel index)*		520 (23.4)	
Yes ( > = 65)		156	30.0%
No (< 65)		364	70.0%
*Delirium (IAGeD)*		535 (21.2)	
No ( < = 3)		478	89.3%
Yes (> 3)		57	10.7%
*Cognitive impairment (MDSCPS)*		567 (16.5)	
Intact (0)		108	19.0%
Borderline intact/Mild (1/2)		134	23.6%
Moderate/Moderately severe (3/4)		181	31.9%
Severe/Very severe (5/6)		144	25.4%
*Comorbidities (Elixhauser)*		545 (19.7)	
No (0/1)		104	19.1%
Yes (2+)		441	80.9%
*Potential avoidable emergency hospital admission (last 12 months)*		679 (0.0)	
No		568	83.7%
Yes		111	16.3%
**Care-home level characteristics**			
*Type*		679 (0.0)	
Residential home		327	48.2%
Nursing home		352	51.8%
*Quality rating*		679 (0.0)	
‘Requires improvement’		111	16.3%
‘Good’		497	73.2%
‘Outstanding’		71	10.5%
Size (number of beds): mean (s.d.)		679 (0.0)	54.3 (16.65)
Occupancy rate (%): mean (s.d.)		639 (5.9)	88.0 (13.04)
Self-funding rate (%): mean (s.d.)		594 (12.5)	49.0 (27.76)

Notes: s.d. = Standard deviation. Residents are from *n* = 34 care homes

### Discriminant validity analysis

Table 4 presents the results of the three multi-level regressions of QoL, as measured by ASCOT-Proxy-Resident, ICECAP-O and EQ-5D-5L Proxy 2. Each column in the table presents the coefficient and standard error from the estimation of the models of the respective measures of QoL, with both level 1, i.e. (demeaned) resident, and level 2, i.e. care home, characteristics included in the models. The latter includes both (care home) means of the resident-level predictors and care home-level predictors. Likelihood-ratio tests for all three QoL measures found there was significant evidence of variation between care homes (ρ < 0.01 for all 100 imputations), confirming the multi-level modelling strategy as appropriate. We found no evidence that the random errors generated by MI significantly affected the findings for any of the QoL regression models, confirming reproducibility if the MI process was repeated.

**Table 4:** Multi-level regression models predicting QoL (*n* = 679)

Parameters	Model 1		Model 2		Model 3	
	Ascot-Proxy-Resident		ICECAP-O		EQ-5D-5L Proxy 2	
	Coef.	S.E.	Coef.	S.E.	Coef.	S.E.
**Resident variables**						
Sex: Male	0.011	0.017	-0.030	0.017	0.049*	0.023
Age: 80–89	0.026	0.018	0.018	0.019	-0.007	0.026
Age: 90+	0.040*	0.020	0.016	0.019	-0.029	0.026
Length of stay: >=3 years	-0.023	0.018	-0.027	0.018	-0.107***	0.021
Functional independence: No	-0.021	0.016	-0.064***	0.015	-0.342***	0.042
Delirium: Yes	-0.067*	0.031	-0.057*	0.026	-0.007	0.033
Cognitive impairment: Borderline intact/Mild	-0.037*	0.017	-0.028	0.016	-0.020	0.025
Cognitive impairment: Moderate/Moderate Severe	-0.078**	0.023	-0.075***	0.021	-0.038	0.030
Cognitive impairment: Severe/Very severe	-0.081**	0.025	-0.172***	0.023	-0.177***	0.037
Comorbidities: Yes	0.008	0.019	-0.008	0.020	-0.034	0.028
Potentially avoidable emergency hospital Admission: Yes	-0.044*	0.020	-0.001	0.019	-0.043	0.029
**Care home variables**						
Sex: Male (Mean)	0.160	0.159	-0.190	0.246	0.159	0.248
Age: 80–89 (Mean)	-0.091	0.160	-0.122	0.226	0.648**	0.206
Age: 90+ (Mean)	0.148	0.142	0.105	0.174	-0.097	0.183
Length of stay: >=3 years (Mean)	-0.011	0.121	-0.062	0.182	0.334	0.179
Functional independence: No (Mean)	-0.091	0.067	-0.132	0.068	-0.527***	0.135
Delirium: Yes (Mean)	0.160	0.146	-0.049	0.167	-0.221	0.173
Cognitive impairment: Borderline intact/Mild (Mean)	0.098	0.161	-0.006	0.155	0.326	0.195
Cognitive impairment: Moderate/Moderate Severe (Mean)	-0.056	0.096	0.028	0.120	0.343**	0.125
Cognitive impairment: Severe/Very severe (Mean)	-0.250	0.133	-0.212	0.138	-0.188	0.145
Comorbidities: Yes (Mean)	-0.023	0.104	0.049	0.131	-0.221	0.170
Potentially avoidable emergency hospital Admission: Yes (Mean)	-0.098	0.196	0.171	0.171	0.064	0.196
Type: Nursing home	-0.090*	0.035	-0.126*	0.040	-0.078	0.049
Quality rating: Good	-0.023	0.050	-0.027	0.068	0.022	0.077
Quality rating: Outstanding	0.113*	0.056	0.134	0.085	0.036	0.089
Size (number of beds)	0.001	0.001	0.0017	0.001	0.0003	0.002
Occupancy rate	-0.0026*	0.001	-0.0002	0.001	0.0001	0.001
Self-funding rate	-0.001	0.001	-0.001	0.001	0.0028*	0.0014
Number of imputations	100		100		100	
Relative variance inflation (RVI)	0.614		0.508		0.597	
Fraction of missing information (FMI)	0.497		0.356		0.685	

Note: ^*^p <.05,^**^p <.01,^***^p <.001. Reference categories are Sex: female; Age: 65–79; Length of stay: <3 years; Functional independence: Yes; Delirium: No; Cognitive impairment: Intact; Emergency admission: No; Comorbidities: No; Type: Residential home; Quality rating: Requires improvement

ASCOT-Proxy-Resident was significantly negatively influenced by level of cognitive impairment and previous potentially avoidable emergency hospital admission and positively influenced by living in a care home with an ‘Outstanding’ CQC quality rating. There was no significant influence of functional dependence on ASCOT-Proxy-Resident. In contrast, ICECAP-O was significantly negatively influenced by functional dependence, and also by level of cognitive impairment. Whereas ASCOT-Proxy-Resident could discern a difference in score at lower levels of impairment, ICECAP-O had differences in score between higher levels of cognitive impairment relative to having no impairment. In particular, within a care home, the average resident with moderate/moderately severe and severe/very severe impairment had a 0.075 (10.2% of average score) and 0.172 (23.3%) lower ICECAP-O score than those with no impairment, respectively.

EQ-5D-5L Proxy 2 was negatively influenced by functional dependence and the highest level of cognitive impairment, but not by previous potentially avoidable emergency hospital admission. The size of influence of functional dependence, for the average resident within a care home, was greatest on EQ-5D-5L Proxy 2 (0.342 lower score, equivalent to 100% of average score) compared to both ICECAP-O (0.064, 8.7% of average score) and ASCOT-Proxy-Resident (not significantly different from zero).

ASCOT-Proxy-Resident and ICECAP-O scores were significantly lower for those residents with delirium and for residents living in nursing homes. The former was also lower for residents over 90 years old and for residents living in homes with higher occupancy levels. EQ-5D-5L Proxy 2 was significantly lower for women and residents with longer length of stays. For EQ-5D-5L, there were also between-care home level associations, with average resident functional dependence, moderate and moderate severe cognitive impairment and for residents aged 80–89 years having a positive influence on health-related QoL between care homes.

### Construct validity by hypothesis testing

Evaluation of the hypotheses concerning discriminant validity of the QoL scores are presented in Table 1. There was sufficient evidence of construct validity for the three QoL measures as > = 75% of hypotheses were accepted for each measure.

## Discussion

This study looked to assess the construct validity of three different QoL measures that could be used in economic evaluation in care homes, namely the ASCOT-Proxy-Resident, ICECAP-O and EQ-5D-5L Proxy 2. In particular, discriminant construct validity was explored using hypothesis testing to understand factors associated with the QoL of care home residents in England. The results support the construct validity of the measures. The findings showed that each measure is associated with different factors contributing to QoL, as expected by measure construct. EQ-5D-5L Proxy 2 was most strongly associated with dependence in performing activities of daily living and also associated with the highest level of cognitive impairment, but not avoidable hospital admissions or comorbidities. A lack of association with cognitive function in older people has been previously established internationally [26–27] but, for England, a different proxy version of the measure also found QoL to be associated with higher levels of cognitive impairment [25]. Also, whilst EQ-5D-5L is well established as a measure of health-related QoL, the Proxy 2 version did not differentiate between care home residents’ QoL based on comorbidity.

As such, for care home studies, particularly those looking to establish the impact of interventions on overall wellbeing, it would be beneficial to measure QoL more broadly than health-related QoL, using the ASCOT-Proxy-Resident and ICECAP-O. ICECAP-O can differentiate between residents by level of functional dependence; both measures were able to differentiate residents by level of impairment and also between residents with and without delirium. Further, ASCOT-Proxy-Resident can differentiate by level of care quality and effectiveness indicators, whilst controlling for functional dependence. This is both at the care home level, as in previous analyses using ASCOT CH4 [16, 18], and also at resident level, with a negative association between QoL and potentially avoidable emergency hospital admissions. The findings as a whole are in line with previous evidence for different versions of ASCOT and ICECAP-O [8, 16, 18–21], although, for the latter measure, the evidence to date is for people living in the community. For ASCOT-Proxy-Resident, having previously been used with family carer proxy report in community settings [66], the DACHA study is the first time that this measure has been used within care homes in England and supports its use in this context with staff proxy report.

Overall, this study has added to the literature by finding that the three instruments of QoL have acceptable construct validity for use in care homes. This adds to other evidence from the DACHA study on the psychometric properties of the measures [17, 28]. The findings also provide evidence that different personal and care home factors explain variation in QoL when measured using the three instruments. This supports the concept that each measure captures different constructs, indicating that the measures may be complementary rather than duplicative. Further research is required on the complementarity of the three QoL measures in care home settings, which would add to the existing evidence base for the measures’ complementarity in community settings [13, 15].

The study has also added to the literature by assessing the association between resident QoL and potentially avoidable emergency hospital admissions. Hospital admissions are likely for care home residents [33] and decisions behind them are complicated, being affected by several factors [67]. However, we expect that, other things equal, an admission classed as potentially avoidable would be more likely to be indicative of some form of unmet need for a care home resident, be that health- or social care-related. We found that ASCOT-Proxy-Resident is sensitive to hospital admissions, but not EQ-5D-5L Proxy 2 (or ICECAP-O). This is tangential to previous research for home care where the significant service impact on QoL was captured by self-report ASCOT-SCT4, but not by self-report EQ-5D-3L [11]. The modelling strategy used in this study also confirms that the finding is not an indicator of care home-level quality, but rather relates to individual-level care in relation to a resident’s fluctuating needs. Overall, an association between QoL and hospital admissions could have important implications for improving outcomes. For example, QoL could be used to predict hospital admissions [68–69]. However, more work is needed to analyse the impact that appropriate health care utilisation has on care home resident QoL using a longitudinal approach.

Further, the findings are in line with previous recommendations that economic evaluations of older people, including those living in care homes, should use different, complementary, QoL measures to consider different constructs, e.g. health-related and social care-related QoL [13–14]. Our findings here support the inclusion of multiple measures of different constructs (health-related QoL, social care-related QoL, capability wellbeing) in a future MDS, subject to the additional data burden for care home providers and their staff [70].

The study used data from the DACHA pilot MDS for care home residents, which linked care home records with health care and administrative data, and specifically included the collection of resident QoL. Currently, nationwide collection of care home resident QoL data is restricted to a survey of those that are publicly-funded and can self-report, therefore missing a large majority of this population. However, government policy is driving a digitalisation agenda which includes the use of DCRs by social care providers to enable better linkage with health care records [34, 70]. As such, a future MDS for care home residents in England is foreseeable. If so, it must have consistent data and be of value to residents [71]. Data in a future MDS would enable research to inform national, local and provider policy and care delivery to improve resident QoL. Importantly, with the three instruments used in this study, this could include economic evaluations.

There are a number of limitations to this study. First, the findings should not be seen as representative of the English care home resident population, although for ASCOT-Proxy-Resident, the profile of social care-related QoL is in line with past research that used a mixed-methods approach to collect social care-related QoL (known as ASCOT-CH4) [16, 18], and they are also similar to findings for ICECAP-O and EQ-5D-5L internationally [19, 20, 21, 26–27]. Second, we were unable to assess changes to QoL over time due to issues with data quality in a second wave of data collection for the pilot MDS [41]. The level of needs of a care home resident are likely to increase over time [72], and it would be of interest to assess how QoL changes with this. Currently, longitudinal analysis is limited in England to those living in their own homes [73–74] or a specific group of residents [75]. A longitudinal analysis would also help mitigate concerns of bias due to omitted variables, which, given the statistical methods employed, was still possible at the resident level [64]. Finally, there were no homes with an overall CQC rating rated as ‘Inadequate’ included in the study. However, the ‘Inadequate’ rating is transitory in nature, with care homes having to improve or face closure [76].

## Conclusion

This study has found that the ASCOT-Proxy-Resident, ICECAP-O and EQ-5D-5L Proxy 2 have acceptable construct validity for use in care homes. Findings also support the concept that the measures are complementary, being based on different constructs. In so doing, the study has provided evidence for the inclusion of these three QoL measures in any future MDS for care home residents.

## Electronic supplementary material

Below is the link to the electronic supplementary material.

Supplementary Material 1

## Data Availability

Anonymised data extracted from digital care records will be available on request from the corresponding author, SA, following a 24 month embargo from the date of publication.

## Abbreviations

ADL: Activity of Daily Living
ASCOT: Adult Social Care Outcomes Toolkit
CQC: Care Quality Commission
DACHA: Developing resources And minimum data set for Care Homes’ Adoption
EQ-5D: EuroQol five-dimension questionnaire
IAGeD: Informant Assessment of Geriatric Delirium Scale
ICECAP-O: Icepop Capability measure for older people
MAR: Missing at random
MCAR: Missing completely at random
MDS: Minimum data set
MDSCPS: Minimum Data Set Cognitive Performance Scale
MI: Multiple imputation
QALY: Quality-adjusted life year
QoL: Quality of life
SCRQoL: Social care-related quality of life
STROBE: Strengthening Reporting of Observational Studies in Epidemiology
