# Supplementary material for: Construct validity of measures of care home resident quality of life: cross-sectional analysis using data from a pilot minimum data set in England
Source: Health Qual Life Outcomes. 2025 Apr 5;23:33. doi: 10.1186/s12955-025-02356-0 (PMC11972536; doi:10.1186/s12955-025-02356-0)
Supplement: Supplementary file 1 — Supplementary Material 1 [file 12955_2025_2356_MOESM1_ESM.docx]

Supplementary Table 1: List of potentially avoidable hospital admissions

| **Primary diagnosis on admission** |
| --- |
| Acute lower respiratory tract infections, e.g. acute bronchitis |
| Chronic lower respiratory tract infections, e.g. emphysema |
| Diabetes |
| Food and drink issues, e.g. abnormal weight loss, poor intake of food and water. |
| Fractures and sprains |
| Intestinal infections |
| Pneumonia |
| Pneumonitis caused by inhaled food or liquid |
| Pressure sores |
| Urinary tract infections |

Source: Lloyd, T., Wolters, A., and Steventon, A. (2017) The impact of providing enhanced support for care home residents in Rushcliffe: Health Foundation consideration of findings from the Improvement Analytics Unit. London: Health Foundation. Available at: <https://www.health.org.uk/reports-and-analysis/briefings/the-impact-of-providing-enhanced-support-for-care-home-residents-in> (accessed 22 January 2025).
